# Therapeutics

**Published:** 1877-09

**Authors:** 


### III. Therapeutics.

A Large Dose of Croton Oil. Sharp. (*The Ohio Med-Record*, May, 1877.) A lady, through mistake, swallowed about a teaspoonful of croton-oil. Discovering the accident, she alarmed the family, who immediately summoned medical assistance. Free administration of sweet milk and cream, and olive oil, in connection with white of eggs and an emetic dose of mustard was given, and supplemented by large doses of sulphate of zinc at short intervals. Patient was also allowed to drink freely of mucilage of gum acaciae. She recovered rapidly, with no ill effects from the oil. W. F. L.

Woorara in Tetanus. Knecht. (*Translated by Watson, N. Y. Med. Jour.*, June 1877.) Dr. K., physician to the prison at Walhiem, has collected a record of fifteen cases of Tetanus treated with Woorara. Two of the number were Tetanus Rheumaticus, and thirteen were Tetanus Traumaticus. The two former recovered, and seven out of the thirteen of the latter. The remedy was administered largely subcutaneously, but a few cases received it also by the mouth.

With some of the cases, chloral was given at the same time, but the greater number were treated by Woorara alone.

The dose by injection was $\frac{1}{125}$th of a grain, which was gradually increased to $\frac{1}{6}$th of a grain, no effect being discovered until the latter dose was reached, after which a remission of the trismus was perceptible.

The maximum dose was $2\frac{1}{3}$ grains. The usual dose was $\frac{1}{2}$ a grain, repeated from two to five or seven times daily, according to the severity of the case.

The greatest amount received by one patient during the course of treatment, was $18\frac{3}{4}$ grains. In this case a condition of general paralysis was produced, with cessation of respiration, which was controlled by applications of electricity, after which improvement rapidly progressed to recovery.

LIST OF PHYSICIANS, in the State and city, who have been licensed to practice medicine by the State Board of Health, to date:

Drs. W. H. Byford, graduated Ohio Medical College, Cincinnati, March 4, 1844; Reuben Ludlam, Medical Department University of Pennsylvania, March, 1851; Robt. L. Rea, Medical College of Ohio, Cincinnati, March 1, 1855; John H. Rauch, Medical Department University of Pennsylvania, March 20, 1849; Ralph E. Starkweather, College of Physicians and Surgeons, N. Y. City, Nov. 10, 1868; Charles C. Buckley, College of Physicians and Surgeons, N. Y. City, March 12, 1867; Charles H. Vilas, Hahnnemann Medical College, Chicago, March 14, 1873; Eddy Bert, University of Göttengen, Germany, Dec. 23, 1864; Samuel Ingraham, Bennett Medical College, Chicago, June 20, 1877; Henry M. Bannister, Columbian College, Washington, D. C., June 20, 1871; Joseph S. Lane, Licentiate Rock River Medical Society, Beloit, Wisconsin, March 16, 1847; Francis A. Emmons, Rush Medical College, Chicago, 1863; King Barton Hunter, University of Nashville, Tennessee, Feb., 1877; Brock McVickar, Fairfield Medical College, Fairfield, N. Y., 1831; Robt. Hunter, University of New York, March 5, 1846; Edwin W. Hunter, Rush Medical College, Chicago, Feb. 21, 1877; Ambrose M. Read, Bennett Medical College, Chicago, Feb. 21, 1877: H. D, Garrison, Eclectic Medical Institute, Cincinnati, Ohio, May 12, 1857; Cornelius A. Logan, Miami Medical College of Ohio, Cincinnati, Feb. 28, 1853; Oscar C. DeWolf, Medical Department University of N. Y., March 2, 1858; E. F. Buecking, Bennett Medical College, March 2, 1876; H. E. Hildebrand, Bennett Medical College, June 22, 1877; William P. Peirce, University of City of N. Y., March 6, 1852; Richard N. Foster, Hahnnemann Medical College, Chicago. March 20, 1869; Joel R. Gore, Medical Department University of N. Y., March 8, 1850; Charles Adams, Hahnnemann Medical College, Feb. 26, 1872; Geo. M. Fox, Castleton Medical College, Castleton, Vt., June 26, 1851; D. Q. Sheppers, Rush Medical College, Chicago, Jan. 26, 1866; Sarah Hackett Stevenson, Woman's Hospital Medical College of Chicago, March 2, 1875; Julins T. C. Hoffman, Rush Medical College, Feb. 3, 1868—University of Würzberg, Germany, Aug. 2, 1870; Robert B. Treat, Eclectic Medical Institute of Cincinnati, Ohio, 1847, P. S. Hayes, Rush Medical College, Chicago, Jan. 17, 1872; Justin Hayes, Cleveland Medical College, Cleveland, Ohio, March 10, 1850; Swayne Wickersham, University of Pennsylvania, March, 1855; Joseph B. Rood, Rush Medical College, Chicago, Feb. 5, 1868; J. Owen Hughes, Rush Medical College, Chicago, Jan. 25, 1867; John Geurin, Rush Medical College, Chicago, Feb. 1866; John M. Pilsbury, Jefferson Medical College, March 10, 1865; John E. Gilman, Hahnnemann Medical College, Feb., 1871; Michael T. O'Clery, Rush Medical College; Charles White, Rush Medical College, Feb. 15, 1863; James I. Tucker, Medical Department Harvard College; Alexander Fisher, Fairfield Medical College, March, 1834; Edward A. Ince, Hahnnemann Medical College, Feb. 12, 1877; Jonathan W. Brooks, Jefferson Medical College, March 4, 1835; Wm. F. Kessler, University of Leipsic, Saxony, April. 1847; James S. Stitt, Chicago Medical College, Chicago, March 9, 1869; Stephen O. Richey, Chicago Medical College, March 21, 1876; Emil Schattenfels, Bennett Medical College, May 21, 1874; John G. S. Mohr, University of Copenhagen, Denmark, July 5, 1867; Timothy R. Nute, Dartmouth Medical College, May, 1850; W. H. Woodyatt, Western Homœpathic Medical College, Feb. 27,

1869; John Reid, Jefferson Medical College, Philadelphia, March, 1847; Frederick A. Hess, Rush Medical College, Feb. 19, 1873; William E. Clark, Vermont Medical College, June 10, 1845; T. B. Shanahan, Rush Medical College; Thos. T. Ellis, The Royal College of Physicians, Edinburgh, Scotland, March, 1842; Alex. H. Cooke, University of the City of New York, March 6, 1846; J. Spafford Hunt, Jefferson Medical College, Philadelphia, March 8, 1856; Geo. P. Cunningham, Rush Medical College, Feb. 21, 1877; Julius Otto, The Chicago Medical College, March 21, 1876; Jacob May, Rush Medical College, Feb., 1876; A. W. Woodward, Hahnnemann Medical College, March 25, 1865; Norman Bridge, The Chicago Medical College, March 9, 1868; John F. Williams, The Chicago Medical College, Mareh, 1865; Henry Upton, Rush Medical College, Jan. 25 1867; James H. Bates, The Long Island Medical College, June 24, 1874; William Tracy, Rush Medical College, Feb. 1, 1877; Gustav H. C. Fricke, Rush Medical College, Feb. 3, 1869; P. O'Connell, The Queen's University in Ireland, June 19, 1872; Louis Varges, The University of Gottingen, May 21, 1833; M. B. Carleman, Rush Medical College, Feb. 3, 1869; John Nicolai, Medical Department Lind University, Chicago, March 4, 1861; Joseph F. Beseler. The University of Berlin, Germany, March 14, 1838; John C. Webster, Harvard Medical College, March 13, 1867; G. C. Paoli, Rush Medical College, Jan. 24, 1866; Eugene S. Atwood, Rush Medical College, Feb. 21, 1877; A. F. Weekes, Rush Medical College, Feb. 22, 1849; C. A. Wilbur, The Castleton Medical College, June 20, 1852; Adelia Barlow, Woman's Hospital Medical College, Chicago, Feb. 29, 1876.

